# Cudrania tricuspidata Root Extract Prevents Methylglyoxal-Induced Inflammation and Oxidative Stress via Regulation of the PKC-NOX4 Pathway in Human Kidney Cells

**DOI:** 10.1155/2021/5511881

**Published:** 2021-03-30

**Authors:** Donghee Kim, Jayeon Cheon, Haelim Yoon, Hee-Sook Jun

**Affiliations:** ^1^Lee Gil Ya Cancer and Diabetes Institute, Gachon University, Incheon 21999, Republic of Korea; ^2^College of Pharmacy and Gachon Institute of Pharmaceutical Sciences, Gachon University, Incheon 21936, Republic of Korea; ^3^Gachon Medical Research Institute, Gil Hospital, Incheon 21565, Republic of Korea

## Abstract

Diabetic nephropathy is a microvascular complication induced by diabetes, and methylglyoxal (MGO) is a reactive carbonyl species causing oxidative stress that contributes to the induction of inflammatory response in kidney cells. *Cudrania tricuspidata* (CT), cultivated in Northeast Asia, has been used as traditional medicine for treating various diseases, including neuritis, liver damage, and cancer. In this study, we determined whether a CT root extract (CTRE) can prevent MGO-induced reactive oxygen species (ROS) production and inflammation and assessed underlying mechanisms using a kidney epithelial cell line, HK-2. We observed that CTRE inhibited MGO-induced ROS production. Additionally, CTRE ameliorated the activation of MGO-induced inflammatory signaling pathways such as p38 mitogen-activated protein kinase (MAPK), extracellular signal-regulated kinase (ERK), and c-JUN N-terminal kinase (JNK). Consistent with these results, expressions of p-nuclear factor-kappa B (NF*κ*B) and inflammatory cytokines, tumor necrosis factor-*α*, interleukin- (IL-) 1*β*, and IL-6, were decreased when compared with MGO-only exposed HK-2 cells. CTRE alleviated the MGO-induced decrease in nuclear factor (erythroid-derived 2)-like 2 (Nrf2) and antioxidant enzyme mRNA expressions. MGO induced the expression of NADPH oxidase 4 (NOX4); CTRE pretreatment inhibited this induction. Further studies revealed that the NOX4 expression was inhibited owing to the suppression of MGO-induced protein kinase C (PKC) activation following CTRE treatment. Collectively, our data suggest that CTRE attenuates MGO-induced inflammation and oxidative stress via inhibition of PKC activation and NOX4 expression, as well as upregulating the Nrf2-antioxidant enzyme pathway in HK-2 cells.

## 1. Introduction

Diabetic nephropathy (DN) is one of the most important and common complications of diabetes [1]. Glomerular hypertrophy, matrix protein accumulation, and tubular injury are major pathological features, and eventually, the loss of kidney function. [2, 3]. Chronic hyperglycemia is known to cause and accelerate kidney damage. Hyperglycemia promotes the formation of advanced glycation end products (AGEs), which are known to play an important role in the development of DN.

Methylglyoxal (MGO) is the main precursor of AGEs and can induce intracellular damage by increasing reactive oxygen species (ROS), mitochondrial damage, and inflammation. Therefore, MGO accumulation has been implicated in vascular complications of diabetes and chronic inflammatory diseases, including cardiovascular disease, cancer, cognitive dysfunction, and bone loss [4–10].

*Cudrania tricuspidata* Bureau (CT), which belongs to the Moraceae family, is widely distributed throughout Northeast Asia, including Korea, China, and Japan. It has been used as a traditional medicine for tumors, liver damage, jaundice, contusions, chronic gastritis, rheumatism, neuritis, and inflammation in East Asia [11, 12]. The roots, stems, leaves, and fruits of CT reportedly contain large amounts of phenolic compounds, including flavonoids, xanthones, diterpenoids, alkaloids, and terpenoids [12–14]. In particular, the roots and stems of CT possess the highest total phenol and flavonoid content when compared with that of leaves and fruits, as well as a strong antioxidant and reducing capacity [13]. Cudratricusxanthone A and cudraflavanone A are reported as active components of CT [15, 16], and cudratricusxanthone A reportedly attenuates renal injury in septic mice [17]. In this study, we investigated the effect of the CT root extract (CTRE) on MGO-induced ROS production and expression of inflammatory cytokines in HK-2 cells, a human tubular epithelial cell line, and assessed the underlying molecular mechanisms.

## 2. Materials and Methods

### 2.1. Preparation of Extracts from CT Root (CTRE)

In this study, we used 70% (*v*/*v*) ethanolic extracts of dried CT root (CTRE). Dried CT root (CTR) was purchased from Miryang Kkujippong Farm Cooperative (Gyeongsangnam-do, Korea) and powdered using a mechanical pulverizer. The dried CTR powder (1.0 kg) was subjected to extraction in 70% ethanol for 3 h using a reflux extraction system by KOC Biotech (Daejeon, Republic of Korea). CTRE was dissolved in dimethyl sulfoxide (DMSO; Duchefa Biochemie B.V., Haarlem, Netherlands) at a concentration of 100 mg/mL and then further diluted with a culture medium for the experiment to reach the required concentration.

### 2.2. Cell Culture

The human renal proximal tubular cell line, HK-2, was obtained from American Type Culture Collection (ATCC, Rockville, MD, USA). HK-2 cells were maintained in RPMI 1640 (Welgene, Daegu, Korea) containing 10% fetal bovine serum (FBS, Gibco, Grand Island, NY, USA), 100 U/mL penicillin, and 100 *μ*g/mL streptomycin (Welgene) at 37°C in an atmosphere of 5% CO_2_ in 95% air.

### 2.3. Cell Viability Assay

Cell viability was estimated using a D-Plus™ CCK cell viability assay kit (Dongin LS, Seoul, Korea) in accordance with the manufacturer's protocol. HK-2 cells (3 × 10^3^ cells/well) were seeded in 96-well plates. After 24 h of culture, the cells were treated with CTRE or MGO. To assess toxicity following CTRE treatment, the cells were treated with different concentrations of CTRE (5–40 *μ*g/mL) for different times, ranging between 3 and 24 h. To measure cell viability following MGO treatment, the cells were treated with different concentrations of MGO (0.125–1.0 mM) for 24 h or 0.5 mM MGO for different periods, ranging between 3 and 24 h. Following incubation, the CTRE- or MGO-treated medium was replaced with 100 *μ*L of CCK working solution, and the cells were further incubated for 4 h. The optical density (OD) was measured at 450 nm using a VersaMax Microplate Reader (Molecular Devices, LLC, Sunnyvale, CA, USA). The OD value of control cells was considered to represent 100% viability [18]. For use in subsequent studies, we selected an MGO concentration of 0.5 mM, which showed 50% cell viability, and a CTRE dose of 20 *μ*g/mL, which demonstrated the highest inhibitory effects.

### 2.4. Measurement of Intracellular Reactive Oxygen Species (ROS) Levels

To examine time- and dose-dependent effects of MGO on ROS production, HK-2 cells (1.5 × 10^5^ cells/well) were seeded in 6-well plates and cultured for 24 h. Then, cells were treated with 0.5 mM MGO for different periods (0.5–12 h) or with different concentrations of MGO (0.125–1.0 mM) for 2 h. To determine the inhibitory effect of CTRE on MGO-induced ROS production, cells were incubated with vehicle (DMSO) or 20 *μ*g/mL CTRE for 1 h and then further incubated for 2 h, with or without 0.5 mM MGO. Intracellular ROS levels were measured through flow cytometry using 2,7-dichlorodihydrofluorescein diacetate (DCFH-DA, Invitrogen, San Diego, CA, USA), as described in our previous report [19].

For fluorescence images, HK-2 cells (2 × 10^4^ cells/well) were incubated overnight in the Nunc™ Lab-Tek™ II Chamber Slide™ system (Thermo Scientific, Rochester, NY, USA). The cells were pretreated with vehicle or 20 *μ*g/mL CTRE for 1 h and then further treated with or without 0.5 mM MGO for 2 h. The cells were treated with 10 *μ*M DCFH-DA for 30 min at 37°C. The cells were fixed in 5% neutral buffered formalin (NBF, Sigma, St. Louis, MO, USA) for 20 min at 4°C. The nuclei were stained with 4′,6-diamidino-2-phenylindole (DAPI; Invitrogen) diluted in DPBS (1 : 1000) at 25°C for 5 min. Then, cells were mounted with fluorescence mounting medium (Dako North America, Inc.) and observed under a confocal microscope (LSM700; Carl Zeiss Inc., Oberkochen, Germany).

### 2.5. Western Blotting

HK-2 cells (5 × 10^5^ cells) were incubated in 90 mm dishes for 24 h. Then, the cells were pretreated with vehicle or 20 *μ*g/mL CTRE for 1 h and then further treated with 0.5 mM MGO for 1 h (for evaluation of p-PKC*β*) or 2 h. For inhibition of p-PKC*β* signaling, cells were treated with 1 *μ*M GF 109203X for 2 h before CTRE treatment. Total cellular protein extractions and western blotting were performed as described in our previous report [20]. Primary antibodies used in this study are listed in Supplementary Table 1. Chemiluminescent signals were developed by treating the blot with ECL reagents, Immobilon-Western chemiluminescent HRP substrate (Millipore Corp., Billerica, MA, USA), and visualized using the LAS4000 imaging system (Fujifilm Corp.; Tokyo, Japan). Protein bands were quantified using ImageJ software (National Institutes of Health, Bethesda, MD, USA).

### 2.6. RNA Isolation and Quantitative Real-Time PCR (RT-qPCR)

HK-2 cells (2 × 10^5^ cells) were incubated in 60 mm dishes for 24 h. The cells were pretreated with vehicle or 20 *μ*g/mL CTRE for 1 h and then treated with or without 0.5 mM MGO for 2 h. Total RNA isolation and RT-qPCR were performed as described in our previous report [21]. In brief, total RNA was extracted using RNAiso Plus Reagent (TaKaRa Bio Inc., Shiga, Japan) and 2 *μ*g of total RNA was reverse-transcribed using a PrimeScript™ 1st-strand cDNA synthesis kit (TaKaRa Bio Inc.). RT-qPCR was performed using a reaction mixture comprising SYBR Green Master Mix (TaKaRa Bio Inc.). Results were calculated using the 2^−*ΔΔ*CT^ relative quantification method and normalized to the expression level of cyclophilin B. Primer pair sequences are shown in Supplementary Table 2.

### 2.7. Statistical Analysis

Statistical analysis was performed by one-way analysis of variance (ANOVA) with Tukey's multiple comparison test using GraphPad Prism version 5 (GraphPad Software Inc., San Diego, CA, USA). Data are presented as the mean ± standard error of the mean (SEM). Statistical significance was set at *p* < 0.05.

## 3. Results

### 3.1. CTRE Does Not Affect HK-2 Cell Viability and Morphology

**Figure 1:**
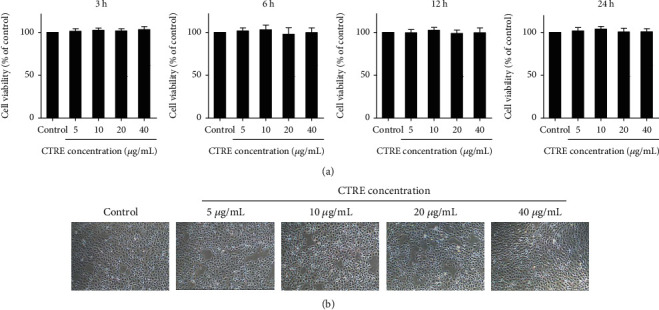
Effect of CTRE on HK-2 cell viability. (a) HK-2 cells were treated with different concentrations (5, 10, 20, and 40 *μ*g/mL) of CTRE for 3, 6, 12, and 24 h. Cell viability was determined using the CCK8 assay kit and calculated relative to that of control cells. Control, vehicle only. Data are presented as the mean ± standard error of the mean (SEM) of three independent experiments. (b) Cell morphology of the control and CTRE groups (5, 10, 20, and 40 *μ*g/mL) treated for 24 h was examined by light microscopy (original magnification, ×40). CTRE: *Cudrania tricuspidata* root extract.

To determine whether CTRE has cytotoxic effects on HK-2 cells, we examined the viability of HK-2 cells after treatment with different concentrations of CTRE for different incubation periods using the CCK assay. On treating HK-2 cells with 5–40 *μ*g/mL CTRE for 3–24 h, no differences in cell viability were observed between the control and CTRE-treated cells at any conditions investigated (Figure 1(a)). As shown in Figure 1(b), the cellular morphology did not differ between the control and 5–40 *μ*g/mL CTRE-treated cells (Figure 1(b)). These results indicate that the CTRE concentrations used in this study did not demonstrate any cellular toxicity in HK-2 cells.

### 3.2. CTRE Inhibits ROS Production in MGO-Treated HK-2 Cells

**Figure 2:**
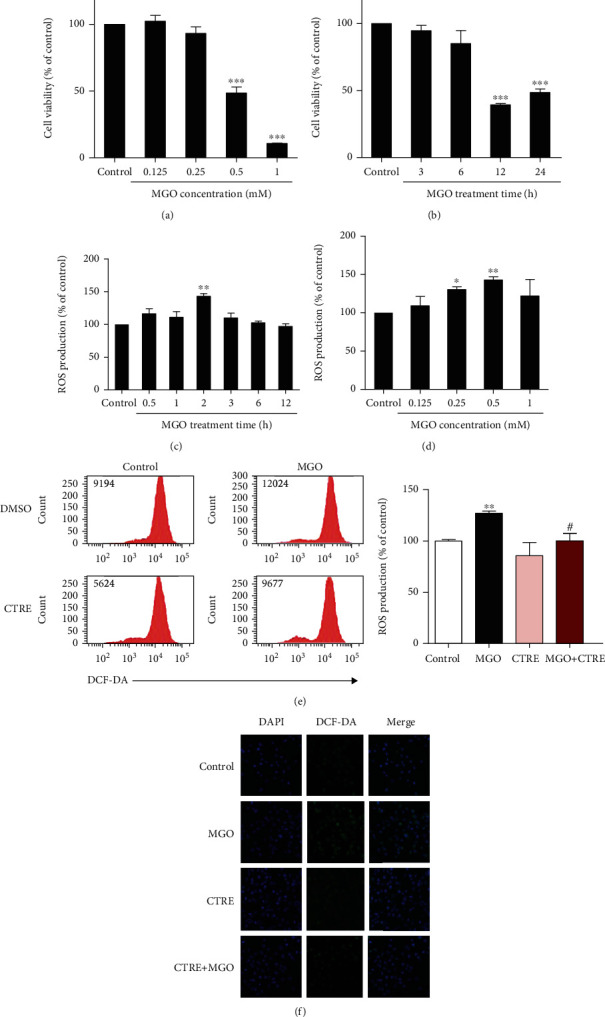
CTRE inhibits ROS production in MGO-treated HK-2 cells. (a, b) Cell viability was determined by the CCK8 assay kit and calculated relative to that of control cells. (a) HK-2 cells were treated with the indicated concentrations of MGO for 24 h. (b) HK-2 cells were treated with 0.5 mM MGO for the indicated times. (c–e) ROS production in cells was measured by flow cytometry with DCFH-DA. (c) HK-2 cells were treated with the 0.5 mM MGO for 0.5–12 h. (d) HK-2 cells were treated with 0.125–1.0 mM MGO for 2 h. (e) HK-2 cells were pretreated with 20 *μ*g/mL CTRE for 1 h and then treated with 0.5 mM MGO for 2 h. Values of the representative flow cytometry data indicate the DCF fluorescence intensity of whole cells. Relative levels of DCF fluorescence intensity was compared with ROS production in the control. Control, vehicle only. Data are presented as the mean ± standard error of the mean (SEM) of three independent experiments. ^∗^*p* < 0.05, ^∗∗^*p* < 0.01, and ^∗∗∗^*p* < 0.005 vs. the control; ^#^*p* < 0.05 vs. MGO. (f) HK-2 cells were seeded on 4-well chamber slide, pretreated with 20 *μ*g/mL CTRE for 1 h, and then treated with 0.5 mM MGO for 2 h. Intracellular ROS levels were determined using confocal microscopy on cells stained with the DCFH-DA (original magnification, ×100). CTRE: *Cudrania tricuspidata* root extract; ROS: reactive oxygen species; MGO: methylglyoxal; DCFH-DA: 2,7-dichlorodihydrofluorescein diacetate.

To determine cell damage induced by MGO, HK-2 cells were treated with 0.125–1.0 mM MGO for 24 h, followed by measurement of cell viability. Cell viability decreased in a dose-dependent manner from 0.5 mM MGO when compared with control cells (Figure 2(a)). Next, HK-2 cells were treated with 0.5 mM MGO, which showed 50% cell viability; when assessed for 3–24 h, cell viability was significantly decreased after 12 h of treatment (Figure 2(b)). As MGO induces cytotoxicity by ROS overproduction in various cell lines [22–25], we measured intracellular ROS levels by flow cytometry in MGO-treated HK-2 cells. As shown in Figure 2(c), ROS production was significantly increased in HK-2 cells treated with 0.5 mM MGO for 2 h compared with that in control cells. On treating HK-2 cells with different concentrations of MGO (0.125–1.0 mM) for 2 h, ROS production was significantly increased when treated with 0.25 and 0.5 mM MGO when compared with that of control cells (Figure 2(d)). We then investigated the effect of CTRE on MGO-induced ROS production and found that pretreatment with CTRE significantly reduced ROS production compared with cells treated with 0.5 mM MGO for 2 h (Figure 2(e)). Confocal image analyses confirmed that MGO greatly increased ROS production, which was strongly attenuated by CTRE pretreatment (Figure 2(f)).

### 3.3. CTRE Decreases the Activation of MAPK and NF*κ*B and the Expression of Inflammatory Cytokines in MGO-Treated HK-2 Cells

**Figure 3:**
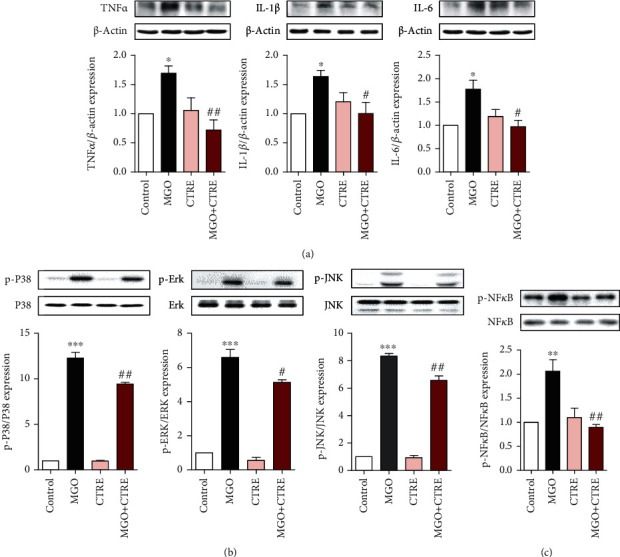
CTRE decreases the expression of inflammatory cytokines and activation of MAPK/NF*κ*B in MGO-treated HK-2 cells. HK-2 cells were pretreated with 20 *μ*g/mL CTRE for 1 h and then treated with 0.5 mM MGO for 2 h. The protein levels of cytokines (TNF*α*, IL-1*β*, and IL-6) (a); p-P38, p-Erk, and p-JNK (b); and p-NF*κ*B (c) were measured by western blotting. The relative expression levels of cytokines and phosphorylated forms were normalized to that of *β*-actin or total forms, respectively, and quantified using ImageJ. Control, vehicle only. Data are presented as the mean ± standard error of the mean (SEM) of three to four independent experiments. ^∗^*p* < 0.05, ^∗∗∗^*p* < 0.005 vs. the control; ^#^*p* < 0.05, ^##^*p* < 0.01 vs. MGO. CTRE: *Cudrania tricuspidata* root extract; MGO: methylglyoxal; MAPK: mitogen-activated protein kinase; NF*κ*B: nuclear factor-kappa B; JNK: c-JUN N-terminal kinase; TNF*α*: tumor necrosis factor *α*; IL-1*β*: interleukin-1*β*; IL-6: interleukin-6.

As MGO-induced oxidative stress activates mitogen-activated protein kinase (MAPK)/nuclear factor-kappa B (NF*κ*B) signaling and induces an inflammatory response [26–28], we examined the expression of inflammatory cytokines in HK-2 cells treated with 0.5 mM MGO in the presence or absence of CTRE. MGO significantly increased the expression of interleukin- (IL-) 1*β*, IL-6, and tumor necrosis factor (TNF) *α* when compared with that in control cells; however, CTRE pretreatment significantly reduced the expression of these genes when compared with that observed in MGO-only exposed cells (Figure 3(a)). We investigated whether the inhibition of MGO-induced cytokine expression by CTRE was mediated via the regulation of MAPK/NF*κ*B activation. Accordingly, we examined the phosphorylation of MAPKs and NF*κ*B in HK-2 cells treated with 0.5 mM MGO, with or without CTRE pretreatment. As shown in Figure 3(b), MGO significantly increased the phosphorylation of P38, extracellular signal-regulated kinase (ERK), and c-JUN N-terminal kinase (JNK) when compared with that in control cells (Figure 3(b)). However, CTRE pretreatment significantly reduced the phosphorylation of these proteins when compared with that in MGO-treated cells (Figure 3(b)). Furthermore, MGO-induced phosphorylation of NF*κ*B was significantly reduced by CTRE pretreatment (Figure 3(c)). These results suggest that CTRE may inhibit the inflammatory cytokine expression by downregulating MAPK/NF*κ*B activation.

### 3.4. CTRE Recovers MGO-Induced Decrease in Nrf2 and mRNA Expressions of Antioxidant Enzymes

**Figure 4:**
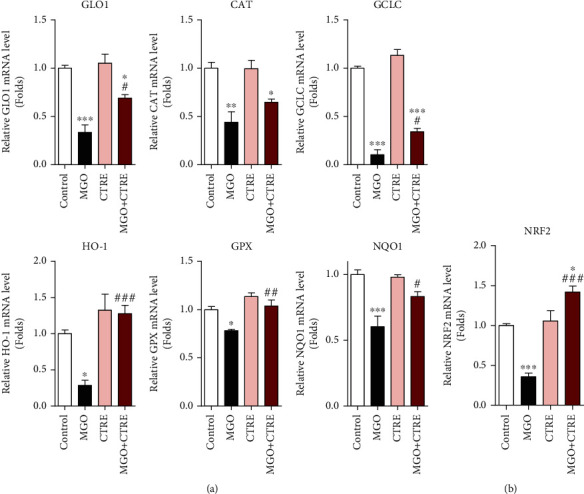
CTRE recovers the MGO-induced decrease in Nrf2 and mRNA expressions of antioxidant enzymes. HK-2 cells were pretreated with 20 *μ*g/mL CTRE for 1 h and then treated with 0.5 mM MGO for 2 h. The mRNA levels of antioxidant enzymes (a) and Nrf2 (b) were measured by qRT-PCR. Control, vehicle only. Data are presented as the mean ± standard error of the mean (SEM) of four independent experiments. ^∗^*p* < 0.05, ^∗∗^*p* < 0.01, and ^∗∗∗^*p* < 0.005 vs. the control; ^#^*p* < 0.05, ^##^*p* < 0.01, and ^###^*p* < 0.005 vs. MGO. CTRE: *Cudrania tricuspidata* root extract; MGO: methylglyoxal; Nrf2: nuclear factor (erythroid-derived 2)-like 2; qRT-PCR: real-time quantitative reverse transcription PCR; GLO1: glyoxalase 1; CAT: catalase; GCLC: glutamate-cysteine ligase catalytic subunit; HO-1: heme oxygenase-1; GPX: glutathione peroxidase; NQO1: NAD(P)H quinone dehydrogenase.

The transcription factor nuclear factor (erythroid-derived 2)-like 2 (Nrf2) acts as a master regulator of cytoprotective responses, including antioxidant, anti-inflammatory, and detoxification [29, 30]. To investigate whether CTRE affects the expression of Nrf2 and antioxidant enzymes, we examined the mRNA expression of Nrf2 and Nrf2 target antioxidant enzymes in MGO-treated HK-2 cells, with and without CTRE pretreatment. The mRNA expression levels of antioxidant genes such as glyoxalase 1 (GLO1), catalase (CAT), glutamate-cysteine ligase catalytic subunit (GCLC), heme oxygenase-1 (HO-1), glutathione peroxidase (GPX), and NAD(P)H quinone dehydrogenase (NQO)1 were significantly reduced following MGO treatment, and all, except CAT, were significantly increased by CTRE pretreatment when compared with that in the MGO-only exposed cells (Figure 4(a)). Consistent with these results, Nrf2 mRNA expression was also significantly increased when compared with that in the MGO-only exposed cells (Figure 4(b)). These results suggest that CTRE may ameliorate the MGO-induced decrease in the antioxidant enzyme gene expression, contributing to a decrease in ROS production.

### 3.5. CTRE Suppresses NOX4 Expression by Downregulating PKC*β* Phosphorylation in MGO-Treated HK-2 Cells

**Figure 5:**
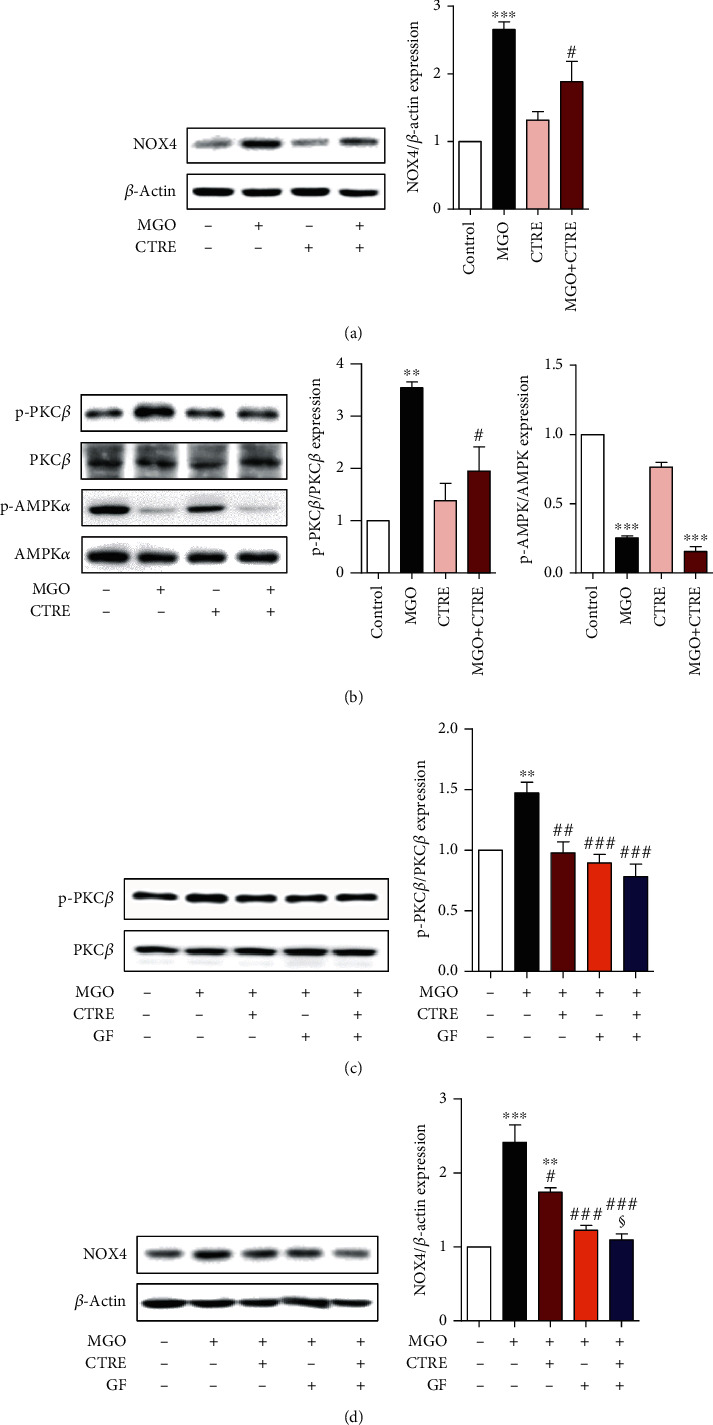
CTRE suppresses NOX4 expression by downregulating PKC*β* phosphorylation in MGO-treated HK-2 cells. (a, b) HK-2 cells were pretreated with 20 *μ*g/mL CTRE for 1 h and then treated 0.5 mM MGO for 2 h (for NOX4, A; for AMPK, B) or 1 h (for PKC*β*, B). (c, d) HK-2 cells were pretreated with or without 1 *μ*M GF 109203X, a PKC inhibitor, for 2 h, and treated with 20 *μ*g/mL of CTRE for 1 h, followed by 0.5 mM MGO treatment for 1 h to detect p-PKC*β* (c) or 2 h to detect NOX4 (d). Protein levels were measured by western blotting. The relative expression levels of NOX4, p-PKC*β*, and p-AMPK*α* were normalized to that of *β*-actin, total PKC*β*, or total AMPK*α*, respectively, and quantified using ImageJ. Control, vehicle only. Data are presented as the mean ± standard error of the mean (SEM) of three to four independent experiments. ^∗∗^*p* < 0.01, ^∗∗∗^*p* < 0.005 vs. the control; ^#^*p* < 0.05, ^##^*p* < 0.01, and ^###^*p* < 0.005 vs. MGO; ^§^*p* < 0.05 vs. MGO+CTRE. CTRE: *Cudrania tricuspidata* root extract; NOX4: NADPH oxidase 4; MGO: methylglyoxal; AMPK: 5′ AMP-activated protein kinase; PKC*β*: protein kinase C beta isoform.

In renal proximal tubular epithelial cells, ROS production is primarily regulated by NOX4 [31–34]. To investigate whether the inhibition of MGO-induced ROS production by CTRE can be attributed to the regulation of NOX4 expression, we measured the protein expression level of NOX4 by western blotting. NOX4 protein levels were significantly increased 2 h after MGO treatment when compared with that in control cells and were significantly reduced by CTRE pretreatment when compared with that in MGO-treated cells (Figure 5(a)). As PKC, particularly PCK*β*, and AMPK pathways are involved in the induction of NOX4 in tubular cells [35], we measured the phosphorylation of PCK*β* or AMPK*α* in the absence or presence of CTRE in MGO-treated HK-2 cells. The phosphorylation of PKC*β* was significantly increased in MGO-treated cells when compared with that in control cells and was significantly reduced following CTRE pretreatment when compared with that in MGO-treated cells (Figure 5(b)). Conversely, MGO reduced the phosphorylation of AMPK*α* when compared with that in control cells; however, CTRE pretreatment did not inhibit the MGO-induced decrease of phosphorylated-AMPK*α* in HK-2 cells (Figure 5(b)). To examine whether p-PKC*β* regulates NOX4 expression, HK-2 cells were treated with GF 109203X (PKC inhibitor) and/or CTRE and then treated with MGO. We observed that pretreatment with GF 109203X significantly inhibited PKC*β* phosphorylation and blocked the expression of NOX4 induced by MGO treatment (Figures 5(c) and 5(d)). Furthermore, we observed additive effects on the inhibition of NOX4 expression following cotreatment with CTRE and GF 109203X (Figure 5(d)). These results suggest that the antioxidant effect of CTRE is caused by the downregulation of NOX4 and ROS production through the inhibition of PKC*β* phosphorylation.

## 4. Discussion

DN is a major complication of diabetes and a common cause of end-stage renal disease. Although DN contributes to the overall morbidity and mortality in diabetes patients, the pathophysiological mechanisms of DN remain unclear [36, 37]. A multidisciplinary therapeutic approach, including renin-angiotensin system (RAS) inhibitors, has long been used to treat DN. The efficacy of sodium-glucose cotransporter 2 (SGLT2) inhibitors has been recently proven and thus added as a new treatment option; however, current therapeutic approaches still do not completely inhibit DN progression [38].

During the progression of DN, glomerular changes such as mesangial expansion, glomerular basement membrane thickening, and podocyte loss are considered clinical signs; however, tubular interstitial inflammation and tubular damage are also critical features [2, 39, 40]. In particular, early changes in tubular epithelial cells are considered an important factor in DN progression [40, 41]. Inflammation from tubular epithelial cells can lead to hyperfiltration, matrix expansion, and apoptosis by causing damage to glomerular cells through inflammatory mediators such as cytokines and chemokines [42–44]. Mohamed et al. reported that the suppression of local inflammation through overexpression of anti-inflammatory factors in tubular epithelial cells improved albuminuria and DN in streptozotocin-induced diabetic mice [44]. Therefore, suppression of inflammation in tubular epithelial cells may be an effective prevention strategy against the development of DN.

Medicinal plants and bioactive natural compounds have been used as alternative treatments for kidney diseases, including DN [45–49]. Previous studies have shown that the fruit, leaves, and roots of CT also have various biological activities, including antioxidant [50, 51], anti-inflammatory [52], antiobesity [53], hepatoprotective [54], and neuroprotective [16, 55] effects. It has recently been reported that CT displays antisenescence effects on endothelial cells exposed to high glucose, a major diabetic factor [56]. In addition, renal protective effects have been reported for cudratricusxanthone A, an active component of CT, in cecal ligation and puncture-induced septic mice [17]; however, the effects of CT on DN have not been studied. In this study, we investigated the effects of the ethanolic extract of CT root on MGO-induced oxidative stress and inflammation in HK-2 cells, a human kidney epithelial cell line.

MGO, a highly reactive dicarbonyl compound, is known as the major precursor for advanced glycation end products (AGEs) and is generated as a side-product derived from glycolysis [57]. MGO- and MGO-mediated AGEs are increased in DN patients, and they are important factors in DN progression [58–60]. It is well established that MGO increases oxidative stress and cell damage/death in endothelial cells [61] and several kidney cells including mesangial cells [62, 63], podocytes [64], and tubular epithelial cells [26, 27, 65]. According to several studies [26–28, 35], including the present study, MGO-induced oxidative stress activates MAPK/NF*κ*B signaling and induces an inflammatory response in renal tubular epithelial cells. Our results show that CTRE pretreatment inhibits MGO-induced ROS overproduction, as well as MAPK and NF*κ*B activation and production of inflammatory cytokines, illustrating the antioxidant and anti-inflammatory effects of CTRE. Previous studies have identified cudraxanthone B, cudratricusxanthone A, cudratricusxanthone O, cudraflavanone A, cudraflavanone B, isogentisin, and 8-prenylxanthone as active ingredients that have anti-inflammatory effects in the CT root [12, 16, 66–68]. Therefore, these active ingredients might decrease the MGO-induced inflammatory response in HK-2 cells.

An increase in antioxidant enzymes through the Nrf2-Keap1 pathway is a major antioxidant response [69]. Cudraflavanone A, isolated from CT root bark, has shown anti-inflammatory effects by Nrf2 activation in microglial cells [16], and a CT leaf extract reportedly exerts antioxidant effects through Nrf2 activation in hepatocytes [70]. Cudratricusxanthone A isolated from the CT root has shown renal protective effects by inducing antioxidant enzymes [17]. Similar to these previous reports [16, 17, 70], CTRE also increased expression of Nrf2 and antioxidant enzymes, suggesting that MGO-induced ROS overproduction may be attenuated by CTRE through increased Nrf2-mediated antioxidant enzyme expression.

NADPH oxidases (NOXs) are a major source of ROS in diabetic kidneys and are important mediators of redox signaling in glomerular and tubulointerstitial cells in diabetic conditions [35]. The NOX family has seven isoforms, and the renal system predominantly expresses NOX1, NOX2, and NOX4 isoforms, of which NOX4 is the most abundant isoform [35, 71, 72]. NOX1 and NOX2 are located in the plasma membrane and require activation by the isoform-specific assembly of different subunits; however, NOX4 is located in the ER, has no regulatory subunit, and is constitutively expressed [71, 73]. Thus, NOX4 has been referred to as a “constitutively active” enzyme, and overall ROS production by NOX4 under various stimulation conditions may be directly regulated by its expression level [35]. NOX4 is highly expressed in the renal cortex, which is mainly composed of tubular epithelial cells [74]. Furthermore, the expression of NOX4 was increased in renal proximal tubular epithelial cells under high glucose conditions; however, NOX2 or NOX1 expression was not changed [31]. Therefore, downregulation of NOX4 expression under diabetic conditions can be employed as a strategy to suppress tubular inflammation. In this study, CTRE pretreatment significantly reduced MGO-induced NOX4 expression in HK-2 cells, suggesting that NOX4 downregulation by CTRE contributes to decreased ROS production.

It is known that the PKC*β*/NOX4 and AMPK*α*/NOX4 pathways are major regulatory pathways for ROS production in renal tubular cells under diabetic conditions [31, 35, 75]. According to a previous report [76], inhibition of PKC*β* signaling attenuates diabetes-induced NOX4 expression and oxidative stress, consistent with our results showing that CTRE inhibits MGO-induced PKC*β* activation, subsequently inhibiting NOX4 expression in HK-2 cells. Meanwhile, inactivation of AMPK by diabetic stimuli increases NOX4 expression and subsequent ROS production in mesangial cells [35, 77]. AMPK activation using AMPK activators, such as metformin or AICAR (5-aminoimidazole-4-carboxamide-1-beta-d-riboruranoside), attenuates high glucose-induced NOX4 expression in tubular epithelial cells [78, 79]. Similar to previous study [77], the expression of phosphorylated-AMPK was significantly reduced following MGO treatment; however, no change was observed following CTRE pretreatment. These results indicate that the inhibition of MGO-induced NOX4 expression by CTRE in HK-2 cells can be attributed to the downregulation of PKC*β* activation, but not by the upregulation of AMPK activation.

**Figure 6:**
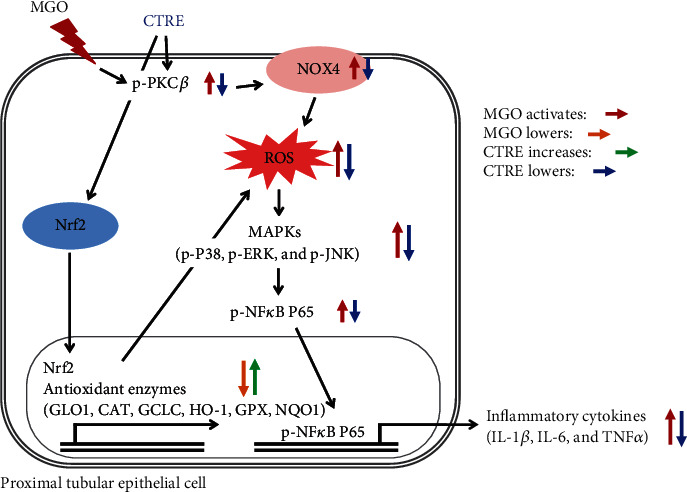
Schematic of the mechanism through which CTRE inhibits MGO-induced tubular cell inflammation and oxidative stress by upregulating the Nrf2-antioxidant enzyme pathway and downregulating the PKC*β*/NOX4 signaling. CTRE: *Cudrania tricuspidata* root extract; MGO: methylglyoxal; NOX4: NADPH oxidase 4; MAPKs: mitogen-activated protein kinases; GLO1: glyoxalase 1; CAT: catalase; GCLC: glutamate-cysteine ligase catalytic subunit; HO-1: heme oxygenase-1; GPX: glutathione peroxidase; NQO1: NAD(P)H quinone dehydrogenase; PKC*β*: protein kinase C beta isoform; ROS: reactive oxygen species; Nrf2: nuclear factor (erythroid-derived 2)-like 2; IL-1*β*: interleukin-1*β*; IL-6: interleukin-6; TNF*α*: tumor necrosis factor *α*.

In summary, our study revealed that CTRE prevents MGO-induced inflammation via attenuation of oxidative stress through inhibition of the PKC-NOX4 pathway, as well as by increasing the Nrf2-antioxidant enzyme pathway in HK-2 cells (Figure 6). Considering the multifactorial and systemic properties of DN and the effects of CTRE on HK-2 cells, we suggest that CTRE or its components might be candidate agents in combination therapy for attenuating chronic inflammation and oxidative stress underlying DN progression.

## Data Availability

The data used to support the findings of this study are available from the corresponding author upon request.

## Supplementary Materials

Supplementary Materials Table S1: antibodies used in the present study. Table S2: primer sequences used for RT-qPCR.

## References

[1] Bhattacharjee N., Barma S., Konwar N., Dewanjee S., Manna P. (2016). Mechanistic insight of diabetic nephropathy and its pharmacotherapeutic targets: an update. *European Journal of Pharmacology*.

[2] Gilbert R. E., Cooper M. E. (1999). The tubulointerstitium in progressive diabetic kidney disease: more than an aftermath of glomerular injury?. *Kidney International*.

[3] Nath K. A. (1992). Tubulointerstitial changes as a major determinant in the progression of renal damage. *American Journal of Kidney Diseases*.

[4] Hanssen N. M. J., Westerink J., Scheijen J. L. J. M. (2018). Higher plasma methylglyoxal levels are associated with incident cardiovascular disease and mortality in individuals with type 2 diabetes. *Diabetes Care*.

[5] Chan C. M., Huang D. Y., Huang Y. P. (2016). Methylglyoxal induces cell death through endoplasmic reticulum stress-associated ROS production and mitochondrial dysfunction. *Journal of Cellular and Molecular Medicine*.

[6] Moraru A., Wiederstein J., Pfaff D. (2018). Elevated levels of the reactive metabolite methylglyoxal recapitulate progression of type 2 diabetes. *Cell Metabolism*.

[7] Bellier J., Nokin M. J., Lardé E. (2019). Methylglyoxal, a potent inducer of AGEs, connects between diabetes and cancer. *Diabetes Research and Clinical Practice*.

[8] Wang H., Liu J., Wu L. (2009). Methylglyoxal-induced mitochondrial dysfunction in vascular smooth muscle cells. *Biochemical Pharmacology*.

[9] Huang X., Wang F., Chen W., Chen Y., Wang N., von Maltzan K. (2012). Possible link between the cognitive dysfunction associated with diabetes mellitus and the neurotoxicity of methylglyoxal. *Brain Research*.

[10] Lee K. M., Lee C. Y., Zhang G., Lyu A., Yue K. K. M. (2019). Methylglyoxal activates osteoclasts through JNK pathway leading to osteoporosis. *Chemico-Biological Interactions*.

[11] Lee I. K., Kim C. J., Song K. S. (1996). Cytotoxic benzyl dihydroflavonols from _Cudrania tricuspidata_. *Phytochemistry*.

[12] Xin L. T., Yue S. J., Fan Y. C. (2017). Cudrania tricuspidata: an updated review on ethnomedicine, phytochemistry and pharmacology. *RSC Advances*.

[13] Park S. Y., Lu G., Kim B., Song W. C., Park G., Choi Y.-W. (2020). A comparative study on physicochemical, photocatalytic, and biological properties of silver nanoparticles formed using extracts of different parts of Cudrania tricuspidata. *Nanomaterials*.

[14] Lee J. S., Han G. C., Han G. P., Nobuyuki K. (2007). The antioxidant activity and total polyphenol content of Cudrania tricuspidata. *Journal of the East Asian Society of Dietary Life*.

[15] Yoon C.-S., Kim D.-C., Quang T. (2016). A prenylated xanthone, cudratricusxanthone a isolated from Cudrania tricuspidata inhibits lipopolysaccharide-induced neuroinflammation through inhibition of NF-*κ*B and p38 MAPK pathways in BV2 microglia. *Molecules*.

[16] Kim K. W., Quang T. H., Ko W. (2018). Anti-neuroinflammatory effects of cudraflavanone a isolated from the chloroform fraction of Cudrania tricuspidata root bark. *Pharmaceutical Biology*.

[17] Lee W., Lee Y., Jeong G.-S., Ku S.-K., Bae J.-S. (2017). Cudratricusxanthone A attenuates renal injury in septic mice. *Food and Chemical Toxicology*.

[18] Cha S.-H., Kim H.-S., Hwang Y., Jeon Y.-J., Jun H.-S. (2018). *Polysiphonia japonica* extract attenuates palmitate-induced toxicity and enhances insulin secretion in pancreatic Beta-cells. *Oxidative Medicine and Cellular Longevity*.

[19] Kim D., Kim H.-J., Cha S.-H., Jun H.-S. (2019). Protective effects of *Broussonetia kazinoki* Siebold fruit extract against palmitate-induced lipotoxicity in Mesangial cells. *Evidence-based Complementary and Alternative Medicine*.

[20] Kim H.-J., Kim D., Yoon H., Choi C. S., Oh Y. S., Jun H.-S. (2020). Prevention of oxidative stress-induced pancreatic beta cell damage by Broussonetia kazinoki Siebold fruit extract via the ERK-Nox4 pathway. *Antioxidants*.

[21] Kim D., Li H. Y., Lee J. H., Oh Y. S., Jun H. S. (2019). Lysophosphatidic acid increases mesangial cell proliferation in models of diabetic nephropathy via Rac1/MAPK/KLF5 signaling. *Experimental & Molecular Medicine*.

[22] Engelbrecht B., Mattern Y., Scheibler S., Tschoepe D., Gawlowski T., Stratmann B. (2014). Methylglyoxal impairs GLUT4 trafficking and leads to increased glucose uptake in L6 myoblasts. *Hormone and Metabolic Research*.

[23] Liu C., Cao B., Zhang Q. (2020). Inhibition of thioredoxin 2 by intracellular methylglyoxal accumulation leads to mitochondrial dysfunction and apoptosis in INS-1 cells. *Endocrine*.

[24] Truong C.-S., Seo E., Jun H.-S. (2019). *Psoralea corylifolia* L. seed extract attenuates methylglyoxal-induced insulin resistance by inhibition of advanced glycation end product formation. *Oxidative Medicine and Cellular Longevity*.

[25] Cha S.-H., Hwang Y., Heo S.-J., Jun H.-S. (2018). Diphlorethohydroxycarmalol attenuates methylglyoxal-induced oxidative stress and advanced glycation end product formation in human kidney cells. *Oxidative Medicine and Cellular Longevity*.

[26] Kim H. Y., Yasuyuki O., Kim M., Yokozawa T. (2008). Protective effect of lipoic acid against methylglyoxal-induced oxidative stress in LLC-PK(1) cells. *Journal of Nutritional Science and Vitaminology (Tokyo)*.

[27] Singh J., Chaudhari B. P., Kakkar P. (2017). Baicalin and chrysin mixture imparts cyto-protection against methylglyoxal induced cytotoxicity and diabetic tubular injury by modulating RAGE, oxidative stress and inflammation. *Environmental Toxicology and Pharmacology*.

[28] Zhao Y., Banerjee S., LeJeune W. S., Choudhary S., Tilton R. G. (2011). NF-*κ*B-inducing kinase increases renal tubule epithelial inflammation associated with diabetes. *Experimental Diabetes Research*.

[29] Theodore M., Kawai Y., Yang J. (2008). Multiple nuclear localization signals function in the nuclear import of the transcription factor Nrf2. *The Journal of Biological Chemistry*.

[30] Pall M. L., Levine S. (2015). Nrf2, a master regulator of detoxification and also antioxidant, anti-inflammatory and other cytoprotective mechanisms, is raised by health promoting factors. *Sheng Li Xue Bao*.

[31] Sedeek M., Callera G., Montezano A. (2010). Critical role of Nox4-based NADPH oxidase in glucose-induced oxidative stress in the kidney: implications in type 2 diabetic nephropathy. *American Journal of Physiology. Renal Physiology*.

[32] Sedeek M., Nasrallah R., Touyz R. M., Hébert R. L. (2013). NADPH oxidases, reactive oxygen species, and the kidney: friend and foe. *Journal of the American Society of Nephrology*.

[33] Geiszt M., Kopp J. B., Varnai P., Leto T. L. (2000). Identification of renox, an NAD(P)H oxidase in kidney. *Proceedings of the National Academy of Sciences of the United States of America*.

[34] Shiose A., Kuroda J., Tsuruya K. (2001). A novel superoxide-producing NAD(P)H oxidase in kidney. *The Journal of Biological Chemistry*.

[35] Gorin Y., Block K. (2013). Nox4 and diabetic nephropathy: with a friend like this, who needs enemies?. *Free Radical Biology & Medicine*.

[36] Himmelfarb J., Tuttle K. R. (2013). New therapies for diabetic kidney disease. *The New England Journal of Medicine*.

[37] Rossing P., de Zeeuw D. (2011). Need for better diabetes treatment for improved renal outcome. *Kidney International. Supplement*.

[38] Yamazaki T., Mimura I., Tanaka T., Nangaku M. (2021). Treatment of diabetic kidney disease: current and future. *Diabetes and Metabolism Journal*.

[39] Gross J. L., De Azevedo M. J., Silveiro S. P., Canani L. H., Caramori M. L., Zelmanovitz T. (2004). Diabetic nephropathy: diagnosis, prevention, and treatment. *Diabetes Care*.

[40] Jheng H. F., Tsai P. J., Chuang Y. L. (2015). Albumin stimulates renal tubular inflammation through an HSP70-TLR4 axis in mice with early diabetic nephropathy. *Disease Models & Mechanisms*.

[41] Morcos M., Sayed A. A. R., Bierhaus A. (2002). Activation of tubular epithelial cells in diabetic nephropathy. *Diabetes*.

[42] Magri C. J., Fava S. (2009). The role of tubular injury in diabetic nephropathy. *European Journal of Internal Medicine*.

[43] Phillips A. O., Steadman R. (2002). Diabetic nephropathy: the central role of renal proximal tubular cells in tubulointerstitial injury. *Histology and Histopathology*.

[44] Mohamed R., Jayakumar C., Ranganathan P. V., Ganapathy V., Ramesh G. (2012). Kidney proximal tubular epithelial-specific overexpression of netrin-1 suppresses inflammation and albuminuria through suppression of COX-2-mediated PGE2 production in streptozotocin-induced diabetic mice. *The American Journal of Pathology*.

[45] Lien E. J., Lien L. L. M., Wang R., Wang J. (2012). Phytochemical analysis of medicinal plants with kidney protective activities. *Chinese Journal of Integrative Medicine*.

[46] Nasri H., Ardalan M. R., Rafieian-Kopaei M. (2014). Mechanistic impacts of medicinal plants in diabetic kidney disease. *Iranian Journal of Public Health*.

[47] Rafieian-Kopaei M. (2013). Medicinal plants for renal injury prevention. *Journal of Renal Injury Prevention*.

[48] Musabayane C. T. (2012). The effects of medicinal plants on renal function and blood pressure in diabetes mellitus. *Cardiovascular Journal of Africa*.

[49] Palipoch S. (2013). A review of oxidative stress in acute kidney injury: protective role of medicinal plants-derived antioxidants. *African Journal of Traditional, Complementary, and Alternative Medicines*.

[50] Lee B. W., Lee J. H., Lee S. T. (2005). Antioxidant and cytotoxic activities of xanthones from _Cudrania tricuspidata_. *Bioorganic & Medicinal Chemistry Letters*.

[51] Lee Y. J., Kim S., Lee S. J., Ham I., Whang W. K. (2009). Antioxidant activities of new flavonoids from Cudrania tricuspidata root bark. *Archives of Pharmacal Research*.

[52] Jeong G. S., Lee D. S., Kim Y. C. (2009). Cudratricusxanthone A from _Cudrania tricuspidata_ suppresses pro-inflammatory mediators through expression of anti-inflammatory heme oxygenase-1 in RAW264.7 macrophages. *International Immunopharmacology*.

[53] Jo Y., Choi K.-M., Liu Q. (2015). Anti-obesity effect of 6,8-diprenylgenistein, an isoflavonoid of Cudrania tricuspidata fruits in high-fat diet-induced obese mice. *Nutrients*.

[54] Tian Y. H., Kim H. C., Cui J. M., Kim Y. C. (2005). Hepatoprotective constituents of Cudrania tricuspidata. *Archives of Pharmacal Research*.

[55] Kwon J., Hiep N. T., Kim D. W. (2016). Chemical constituents isolated from the root bark of Cudrania tricuspidata and their potential neuroprotective effects. *Journal of Natural Products*.

[56] Kim G. D., Park S. (2020). Effects of Cudrania tricuspidata on anti-senescence in high glucose-treated endothelial cells via the Akt/p53/p21 pathway. *Food Science & Nutrition*.

[57] Bourajjaj M., Stehouwer C. D. A., van Hinsbergh V. W. M., Schalkwijk C. G. (2003). Role of methylglyoxal adducts in the development of vascular complications in diabetes mellitus. *Biochemical Society Transactions*.

[58] Fukami K., Yamagishi S. I., Ueda S., Okuda S. (2008). Role of AGEs in diabetic nephropathy. *Current Pharmaceutical Design*.

[59] Rabbani N., Thornalley P. J. (2013). The critical role of methylglyoxal and glyoxalase 1 in diabetic nephropathy. *Diabetes*.

[60] Lu J., Randell E., Han Y. C., Adeli K., Krahn J., Meng Q. H. (2011). Increased plasma methylglyoxal level, inflammation, and vascular endothelial dysfunction in diabetic nephropathy. *Clinical Biochemistry*.

[61] Do M. H., Lee J. H., Ahn J., Hong M. J., Kim J., Kim S. Y. (2020). Isosamidin from Peucedanum japonicum roots prevents methylglyoxal-induced glucotoxicity in human umbilical vein endothelial cells via suppression of ROS-mediated Bax/Bcl-2. *Antioxidants*.

[62] Liu B. F., Miyata S., Hirota Y. (2003). Methylglyoxal induces apoptosis through activation of p38 mitogen-activated protein kinase in rat mesangial cells. *Kidney International*.

[63] Lee J. H., Subedi L., Kim S. Y. (2020). Effect of cysteine on methylglyoxal-induced renal damage in mesangial cells. *Cell*.

[64] Sohn E., Kim J., Kim C. S., Jo K., Kim J. S. (2015). Extract of Rhizoma Polygonum cuspidatum reduces early renal podocyte injury in streptozotocin-induced diabetic rats and its active compound emodin inhibits methylglyoxal-mediated glycation of proteins. *Molecular Medicine Reports*.

[65] Park S.-H., Choi H.-I., Ahn J. (2020). Autophagy functions to prevent methylglyoxal-induced apoptosis in HK-2 cells. *Oxidative Medicine and Cellular Longevity*.

[66] Jo Y. H., Kim S. B., Liu Q., Hwang B. Y., Lee M. K. (2017). Prenylated xanthones from the roots ofCudrania tricuspidataas inhibitors of lipopolysaccharide-stimulated nitric oxide production. *Archiv der Pharmazie*.

[67] Lee W., Ku S.-K., In Kim T. (2021). Inhibitory effects of cudratricusxanthone O on particulate matter-induced pulmonary injury. *International Journal of Environmental Health Research*.

[68] Ko W., Kim K.-W., Quang T. H. (2021). Cudraflavanone B isolated from the root bark of *Cudrania tricuspidata* alleviates lipopolysaccharide-induced inflammatory responses by downregulating NF-*κ*B and ERK MAPK signaling pathways in RAW264.7 macrophages and BV2 microglia. *Inflammation*.

[69] Espinosa-Diez C., Miguel V., Mennerich D. (2015). Antioxidant responses and cellular adjustments to oxidative stress. *Redox Biology*.

[70] Cho S. S., Yang J. H., Seo K. H. (2019). Cudrania tricuspidata extract and its major constituents inhibit oxidative stress-induced liver injury. *Journal of Medicinal Food*.

[71] Bedard K., Krause K. H. (2007). The NOX family of ROS-generating NADPH oxidases: physiology and pathophysiology. *Physiological Reviews*.

[72] Gill P. S., Wilcox C. S. (2006). NADPH oxidases in the kidney. *Antioxidants & Redox Signaling*.

[73] Schulz E., Münzel T. (2008). NOX5, a new "radical" player in human atherosclerosis?^∗^. *Journal of the American College of Cardiology*.

[74] Gorin Y., Block K., Hernandez J. (2005). Nox4 NAD(P)H oxidase mediates hypertrophy and fibronectin expression in the diabetic kidney. *The Journal of Biological Chemistry*.

[75] Chen N. K. F., Chong T. W., Loh H.-L. (2013). Negative regulatory responses to metabolically triggered inflammation impair renal epithelial immunity in diabetes mellitus. *Journal of Molecular Medicine (Berlin, Germany)*.

[76] Ohshiro Y., Ma R. C., Yasuda Y. (2006). Reduction of diabetes-induced oxidative stress, fibrotic cytokine expression, and renal dysfunction in protein kinase Cbeta-null mice. *Diabetes*.

[77] Papadimitriou A., Peixoto E. B. M. I., Silva K. C., Lopes de Faria J. M., Lopes de Faria J. B. (2014). Increase in AMPK brought about by cocoa is renoprotective in experimental diabetes mellitus by reducing NOX4/TGF*β*-1 signaling. *The Journal of Nutritional Biochemistry*.

[78] Lee J. H., Kim J. H., Kim J. S. (2013). AMP-activated protein kinase inhibits TGF-*β*-, angiotensin II-, aldosterone-, high glucose-, and albumin-induced epithelial-mesenchymal transition. *American Journal of Physiology. Renal Physiology*.

[79] Eid A. A., Ford B. M., Block K. (2010). AMP-activated protein kinase (AMPK) negatively regulates Nox4-dependent activation of p53 and epithelial cell apoptosis in diabetes,. *The Journal of Biological Chemistry*.

