# Supplementary material for: Cudrania tricuspidata Root Extract Prevents Methylglyoxal-Induced Inflammation and Oxidative Stress via Regulation of the PKC-NOX4 Pathway in Human Kidney Cells
Source: Oxid Med Cell Longev. 2021 Mar 30;2021:5511881. doi: 10.1155/2021/5511881 (PMC8026309; doi:10.1155/2021/5511881)
Supplement: Supplementary Materials — Table S1: antibodies used in the present study. Table S2: primer sequences used for RT-qPCR. [file 5511881.f1.docx]

**Supplementary Materials**

**Supplemental Table S1: Antibodies used in the present study**

| Antibody | Source | Cat. No. | Dilution |
| --- | --- | --- | --- |
| IL-6 | Abcam | ab9324 | 1:500 |
| NADPH oxidase 4 | Abcam | ab195524 | 1:2000 |
| Phospho-AMPKα (Thr172) | CST | 2535S | 1:2000 |
| AMPKα | CST | 2532S | 1:2000 |
| Phospho-p38 MAPK (Thr180/Tyr182) | CST | 4511S | 1:2000 |
| p38 MAPK | CST | 9212S | 1:2000 |
| Phospho-p44/42 MAPK (Erk1/2) (Thr202/Tyr204) | CST | 9101S | 1:2000 |
| p44/42 MAPK (Erk1/2) | CST | 9102S | 1:2000 |
| Phospho-PKC (pan) (βII Ser660) | CST | 9371S | 1:2000 |
| PKCβ | CST | 46809S | 1:2000 |
| Phospho-SAPK/JNK (Thr183/Tyr185) | CST | 9251S | 1:1000 |
| SAPK/JNK | CST | 9252S | 1:1000 |
| β-actin | Santa Cruz | SC-47778 | 1:2000 |
| IL-1β | Santa Cruz | SC-52012 | 1:500 |
| TNFα | Santa Cruz | SC-1350 | 1:500 |

CST, Cell Signaling Technology; Santa Cruz, Santa Cruz Biotechnology, Inc.

**Supplemental Table S2: Primer sequences used for RT-qPCR**

| Gene name | Sequences 5′-3′ |
| --- | --- |
| *CAT* | Forward 5′-TTT CCC AGG AAG ATC CTG AC-3′ |
|  | Reverse 5′-ACC TTG GTG AGA TCG AAT GG-3′ |
| *GCLC* | Forward 5′-AGT TGA GGC CAA CAT GCG AA-3′ |
|  | Reverse 5′-TGA AGC GAG GGT GCT TGT TT-3′ |
| *GLO1* | Forward 5′-ATG CGA CCC AGA GTT ACC AC-3′ |
|  | Reverse 5′-CCA GGC CTT TCA TTT TAC CA-3′ |
| *GPX* | Forward 5′-AGA ATG TGG CGT CCC TCT GA-3′ |
|  | Reverse 5′-CAG CTC GTT CAT CTG GGT GTA G-3′ |
| *HO-1* | Forward 5′-CAG GCA ATG GCC TAA ACT TC-3′ |
|  | Reverse 5′-GCT GCC ACA TTA GGG TGT CT-3′ |
| *NQO1* | Forward 5′-GTT GCC TGA AAA ATG GGA GA-3′ |
|  | Reverse 5′-AAA AAC CAC CAG TGC CAG TC-3′ |
| *NRF2* | Forward 5′-GAT GGA CTT GGA GTT GCC AC-3′ |
|  | Reverse 5′-AAA GGC CTT CTC CTG TTC CT-3′ |
| *Cyclophilin* | Forward 5′-TGC CAT CGC CAA GGA GTA G-3′ |
|  | Reverse 5′-TGC ACA GAC GGT CAC TCA AA-3′ |
